# Clinical application of multicolor scanning laser ophthalmology in diagnosis and grading of central retinal artery occlusion

**DOI:** 10.3389/fnins.2024.1327806

**Published:** 2024-04-10

**Authors:** Yuwei Wan, Ting Chen, Ying Li, Yang Yang, Yaqi Wang, Yuedan Wang, Xuejie Li, Anhuai Yang, Xuan Xiao

**Affiliations:** Department of Ophthalmology, Renmin Hospital of Wuhan University, Wuhan, Hubei, China

**Keywords:** neurodegenerative disease, multicolor imaging, central retinal artery occlusion, diagnosis, grading, color fundus photography

## Abstract

**Purpose:**

To characterize features of central retinal artery occlusion (CRAO) using multicolor (MC) imaging and to assess the differences in CRAO grading between color fundus photography (CFP) and MC image qualitatively and quantitatively.

**Methods:**

We conducted a prospective, cross-sectional study in the Department of Ophthalmology of Renmin Hospital of Wuhan University. In total, 86 acute CRAO patients were included. Spectral-domain optical coherence tomography (SD-OCT), CFP, and MC examinations were taken at baseline. Based on the findings of these three examinations, CRAO was divided into three grades (incomplete, subtotal, and total). Based on OCT grading criteria, we qualitatively compared the ability of grading CRAO by CFP and MC. CRAO patient's visual acuity (VA) was obtained from the initial visit. The retinal thickness was measured by SD-OCT. Superficial capillary plexus (SCP) and deep capillary plexus (DCP) were obtained from optical coherence tomography angiography (OCTA) examinations. Quantitative data were compared across the three acute CRAO subgroups and against three examination findings.

**Results:**

MC image had significantly higher power of acute CRAO detection than CFP (*P* = 0.03). In the same group of CRAO patients, there was no significant difference in VA when comparing OCT with the MC grading system or with the CFP grading system (all *P* > 0.05). Significant differences in VA were found between the three CRAO subgroups only under MC grading (*P* = 0.016). In incomplete CRAO patients, significant differences were found in central fovea thickness (CFT) when comparing OCT with the CFP grading system (*P* = 0.019). In the same group of CRAO patients, there was no significant difference in retinal thickness when comparing OCT with the MC grading system (All *P* > 0.05). Significance differences in CFT (*P* < 0.001), innermost retinal layer (IMRL; *P* < 0.01), middle retinal layer (MRL; *P* < 0.001), and outer retinal layer (ORL; *P* = 0.021) were found between the three CRAO subgroups by MC grading. Vessel density of SCP showed a statistically increased as the severity of three CRAO subgroups (*P* = 0.03), whereas DCP did not have significant differences (*P* = 0.745). Comparisons were made between the OCT grading method and the MC and CFP grading methods; there is no significant difference in vessel density of SCP and DCP (All *P* > 0.05).

**Conclusion:**

The images obtained by MC are superior to those obtained by CFP in CRAO grading, retinal thickness, and vessel density measurement. MC imaging may be more capable of CRAO grading than OCT. We recommend MC imaging to determine CRAO severity to guide disease treatment and predict visual prognosis.

## 1 Introduction

Central retinal artery occlusion (CRAO) is a vision-threatening eye disease resulting from acute interruption of the central retinal artery. CRAO patients have poor visual recovery, with 90% of patients having final visual acuities of 20/400 or lower. Visual improvement most likely occurs within the first 7 days of CRAO (Hayreh and Zimmerman, 2005). Different stages of CRAO are obviously correlated with visual outcomes (Schmidt et al., 2002; Ahn et al., 2013), thus highlighting the importance of acute CRAO grading in predicting visual prognosis. A host of therapeutic interventions have been tried for visual recovery, while the treatment response among CRAO patients varies individually, and consensus on universally effective treatment modalities remains elusive (Lin et al., 2023). It may be related to the fact that various studies did not differentiate the stage of CRAO correctly, leading to errors in data comparison (Schmidt et al., 2002). A simple and clear grading system of CRAO is needed to help predict visual outcomes and assess the prognostic factors in patients.

The first grading system of CRAO suggested by Schmidt et al. is based on color fundus photography (CFP) combined with fundus fluorescein angiography (FA; Schmidt et al., 2002). According to the degree of vision loss, extent of retinal edema, and delay in arterial blood flow, CRAO was categorized as incomplete, subtotal, or total CRAO. However, some limitations exist. For example, the obstructive arterial and capillary at different retinal layers cannot be accurately estimated by CFP and FA. Determining the extent of retinal edema with CFP is often ambiguous. As an invasive method, FA limits the feasibility of testing for patients with dye allergies and poor liver and kidney function. Moreover, FA only provides information on retinal vascular structures. Thus, a more objective and simple method to determine the CRAO stages would be beneficial. Spectral-domain optical coherence tomography (SD-OCT), which can show the structure of the retina, has been proposed as a useful non-invasive tool for grading retinal ischemia in CRAO (Ahn et al., 2015).

Multicolor (MC) scanning laser ophthalmoscopy imaging is a newly developed imaging modality for Spectralis SD-OCT, capturing the images of different layers of retina and choroid by using blue, green, and infrared wavelengths to produce blue (BR), green (GR), and infrared (IR) reflectance image, respectively (Tan et al., 2016; Feng et al., 2019). These three images were then integrated to form a composite MC image. It allows simultaneous recording of cross-sectional imaging and retinal thickness or fluorescein angiograph and stored in the same instrument. Compared to conventional color fundus images, the merged images can detect pathological changes from different retinal layers with greater clarity (Terasaki et al., 2018). It has been suggested that MC image is superior to CFP in detecting the pathological changes in posterior segment diseases (Ben Moussa et al., 2015; Kilic Muftuoglu et al., 2018; Terasaki et al., 2018; Feng et al., 2019). However, the MC image on CRAO is rarely studied (Venkatesh et al., 2020). In this study, we are interested in determining the associations between MC images and the normal examinations such as CFP, OCT, and optical coherence tomography angiography (OCTA) in CRAO patients. We also try to find out whether MC images can represent CRAO as seen by CFP and contribute to CRAO grading.

## 2 Methods

### 2.1 Study design

Our research was a prospective, cross-sectional study. The study was approved by the Ethical Committee Board of Renmin Hospital of Wuhan University (2020-X-58 and WDRY2022-K278) and adhered to the tenets of Declaration of Helsinki. All patients signed a written informed consent.

### 2.2 Patient selection

We included CRAO patients hospitalized at the Department of Ophthalmology, Renmin Hospital of Wuhan University, from May 2020 to April 2023. The included criteria were as follows: (1) definitive clinical diagnosis of acute CRAO (symptom onset < 14 days; Chen et al., 2015) and (2) had undergone CFP, MC, and SD-OCT examinations on the same day. The excluded criteria were as follows: (1) patients with concurrent other retinal vascular diseases, severe non-proliferative or proliferative diabetic retinopathy, glaucoma, macular disease, giant cell arteritis, or other diseases affecting the results of ocular imaging were excluded; (2) had special treatment prior to obtaining ocular imaging results; and (3) not agree to take part in this study.

### 2.3 Ophthalmic examination

All CRAO patients included in our study underwent comprehensive ophthalmologic evaluations, including visual acuity (VA, converted to logMAR for analysis), slit-lamp biomicroscope, fundus examination, CFP (Digital Fundus Camera, VISUCAM 200; Carl Zeiss Meditec AG, Jena, Germany), MC image, and SD-OCT (OCT Spectralis, Heidelberg Engineering, Heidelberg, Germany). Some patients also had the OCTA (Optovue Inc., Fremont, CA, USA) results.

VA in profound vision loss was assigned as follows: counting finger, 2.3 logMAR; hand movement, 2.5 logMAR; light perception, 2.7 logMAR; and no light perception, 2.9 logMAR (Lange et al., 2009). For OCT examination, the macula was scanned using the “single line” mode, which consisted of a single line of horizontal raster scans centered on the standard nine frames and covered 6 × 6 mm, at a rate of 40,000 A-scans per second. The images were captured when the horizontal planes passed through the fovea. The scan angle of the MC image was 30°, and the wavelengths were 488, 518, and 820 nm for the blue, green, and infrared scanning lasers, respectively. SD-OCT images were analyzed using OCT Spectralis. During the OCTA examination, all participants were examined in a dark room with the pupils in their natural state. The scanning procedure consists of an angio-OCTA 3 × 3 mm scan. To minimize motion artifacts, OCTA utilizes real-time tracking technology.

### 2.4 Classification of disease grades

The acute CRAO was graded according to the classification method suggested by Ahn et al. (2015) which was classified as incomplete, subtotal, and total CRAO based on the specific characteristics of SD-OCT morphology. Inner retinal layers displayed hyperreflectivity in incomplete CRAO, and the retinal structure was clear and intact without retinal thickening. Loss of layer-by-layer structure in the inner retina and increased reflectivity with moderate inner retinal thickness are characteristics of subtotal CRAO. In total CRAO, there were inner retinal layer hyperreflectivity, severe macular edema, a lack of ordered layer structure, and subfoveal choroidal thinning. In the CFP image, incomplete CRAO showed slight retinal ischemic whitening. Moderate ischemic retinal edema with a cherry-red patch is visible in cases of subtotal CRAO. In total CRAO patients, extreme retinal edema and a distinct cherry-red patch were found on CFP (Schmidt et al., 2002). Based on MC images, we conducted a qualitative analysis in order to determine the relationship between multicolor and the severity of CRAO. The MC image grading for CRAO was conducted in two stages. First, three authors (T.C., Y.W., and Y.Y.) discussed and summarized the features of MC images at different stages of CRAO to establish a consensus. Second, two authors (X.L. and Y.L.) used this rule to grade a further larger series of acute CRAO eyes with MC images. To make the results of SD-OCT, MC, and CFP blinded to each other, two authors (X.L. and Y.L.) first assessed the SD-OCT images independently and then the MC images and last the CFP images. Any disagreements were resolved by consensus among authors.

### 2.5 Retinal thickness measurement

The retinal thickness was measured using the built-in distance-measuring tool of OCT software. We measured retinal thickness using Olga's method (Furashova and Matthé, 2017). First, we measured central foveal thickness (CFT). Second, we chose the spot in 1,000 μm temporal of the fovea. Moreover, we calculated the special retinal thickness from this spot. As retinal layer of CRAO patients is more difficult to define when the retinal structure is damaged, we divided the retinal layer measurement into three parts: the innermost retinal layer (IMRL), the middle retinal layer (MRL), and the outer retinal layer (ORL). The retinal nerve fiber layer (RNFL), ganglion cell layer (GC), and inner plexiform layer (IPL) make up the IMRL. The MRL consists of the inner nuclear layer (INL) and the outer plexiform layer (OPL). The retina between the inner border and retinal pigment epithelium (RPE) of the OPL is known as ORL. The layer thickness and segmentation must be as closely assessed in CRAO with retinal structure loss. The retinal thickness was manually and independently measured by two examiners (X.L. and Y.L.).

### 2.6 The vessel density of SCP and DCP measurement

Some CRAO patients underwent OCTA examination. This instrument defaulted the superficial retinal vessel complex located from the inner limiting membrane layer (ILM) to the upper 10 μm of the IPL layer. Moreover, the deep retinal vessel complex comprises the upper 10 μm of the IPL to the lower 10 μm of the OPL. By selecting the stratification of blood flow OCTA with simultaneous display of structure OCT, the proportion of the area of the corresponding region occupied by the blood flow signal was obtained. The vessel density of superficial capillary plexus (SCP) and deep capillary plexus (DCP) were then exported for flow index analysis.

### 2.7 Statistical analysis

Qualitative data were presented as numbers and percentages, and quantitative variables were given as mean ± standard deviation (SD). SD-OCT was used as a gold standard to compare the positive percent agreements of CRAO grading as achieved by MC image and CFP. Calculations of the sensitivity and specificity values were made using CFP as the reference and MC as the test. Values for similarity, sensitivity, and specificity were also evaluated using CFP as the test and MC as the reference. Intraclass correlation coefficient (ICC) for the repeatability of the measurement of retinal thickness by two examiners was calculated. The three CRAO subgroups graded by MC, CFP, and OCT were compared for VA, retinal thickness, SCP, and DCP using a one-way multivariate factorial ANOVA with *post-hoc* Sidak's test. Differences in VA, retinal thickness, SCP, and DCP in acute CRAO patients with different grading modalities were compared two-by-two with independent samples *t*-test. *P*-value < 0.05 was considered to be statistical significance. Statistical analysis was performed using IBM SPSS statistics version 26 (IBM SPSS Statistics; IBM Corporation, Chicago, IL, USA).

## 3 Results

In total, 86 eyes of 86 acute CRAO patients (52 males and 34 females) were included in this study, with a mean age of 59.70 ± 12.43 years (range 25–86 years). The percentage of incomplete, subtotal, and total CRAO patients based on the SD-OCT grading were 7.0% (6/86), 39.5% (34/86), and 53.5% (46/86), respectively. The mean VA of the above CRAO stage was 2.1 ± 0.5 logMAR (range 1.0–2.3 logMAR), 2.3 ± 0.4 logMAR (range 0.9–2.9 logMAR), and 2.5 ± 0.4 logMAR (range 0.2–2.9 logMAR), respectively. Under CFP grading system, the percentage of incomplete, subtotal, and total CRAO patients was 15.1% (13/86), 36.0% (31/86), and 48.8% (42/86), respectively; the mean VA was 2.4 ± 0.3 logMAR (range 1.4–2.9 logMAR) in incomplete CRAO, 2.3 ± 0.4 logMAR (range 0.9–2.9 logMAR) in subtotal CRAO, and 2.4 ± 0.4 logMAR (range 0.2–2.9 logMAR). Moreover, in the MC grading system, the mean VA was 2.0 ± 0.5 logMAR (range 1.0–2.5 logMAR) in incomplete CRAO with a percentage of 11.6% (10/86), 2.4 ± 0.3 logMAR (range 0.9–2.9 logMAR) in subtotal CRAO with a percentage of 33.7% (29/86), and 2.4 ± 0.4 logMAR (range 0.2–2.9 logMAR) in total CRAO with a percentage of 55.0% (47/86). We analyzed VA in different CRAO groups under the MC, OCT, and CFP grading systems. Significant differences in VA were seen between the different CRAO subgroups only under the MC grading system (*P* = 0.016). However, there were no differences in VA between CRAO subgroups under the OCT and CFP grading systems (*P*_OCT_ = 0.078, *P*_CFP_ = 0.313; Table 1). We further compared VA in the same CRAO subgroup under MC, OCT, and CFP grading systems. We found that no significant differences were found between MC images and OCT images as well as CFP and OCT images (all *P* > 0.05; Supplementary Tables 1, 2).

**Table 1:** VA at different grades of CRAO identified by OCT, MC, and CFP.

**VA in different images/ disease grade**	**Incomplete**	**Subtotal**	**Total**	***P*-value**
MC	2.0 ± 0.5	2.4 ± 0.3	2.4 ± 0.4	0.016^*^
OCT	2.1 ± 0.5	2.3 ± 0.4	2.5 ± 0.4	0.078
CFP	2.4 ± 0.3	2.3 ± 0.4	2.4 ± 0.4	0.313

VA, visual acuity; CRAO, central retinal artery occlusion; MC, multicolor; CFP, fundus photography; SD-OCT, spectral-domain optical coherence tomography.

^*^Statistically significant across the three grades of CRAO.

**Figure 1:**
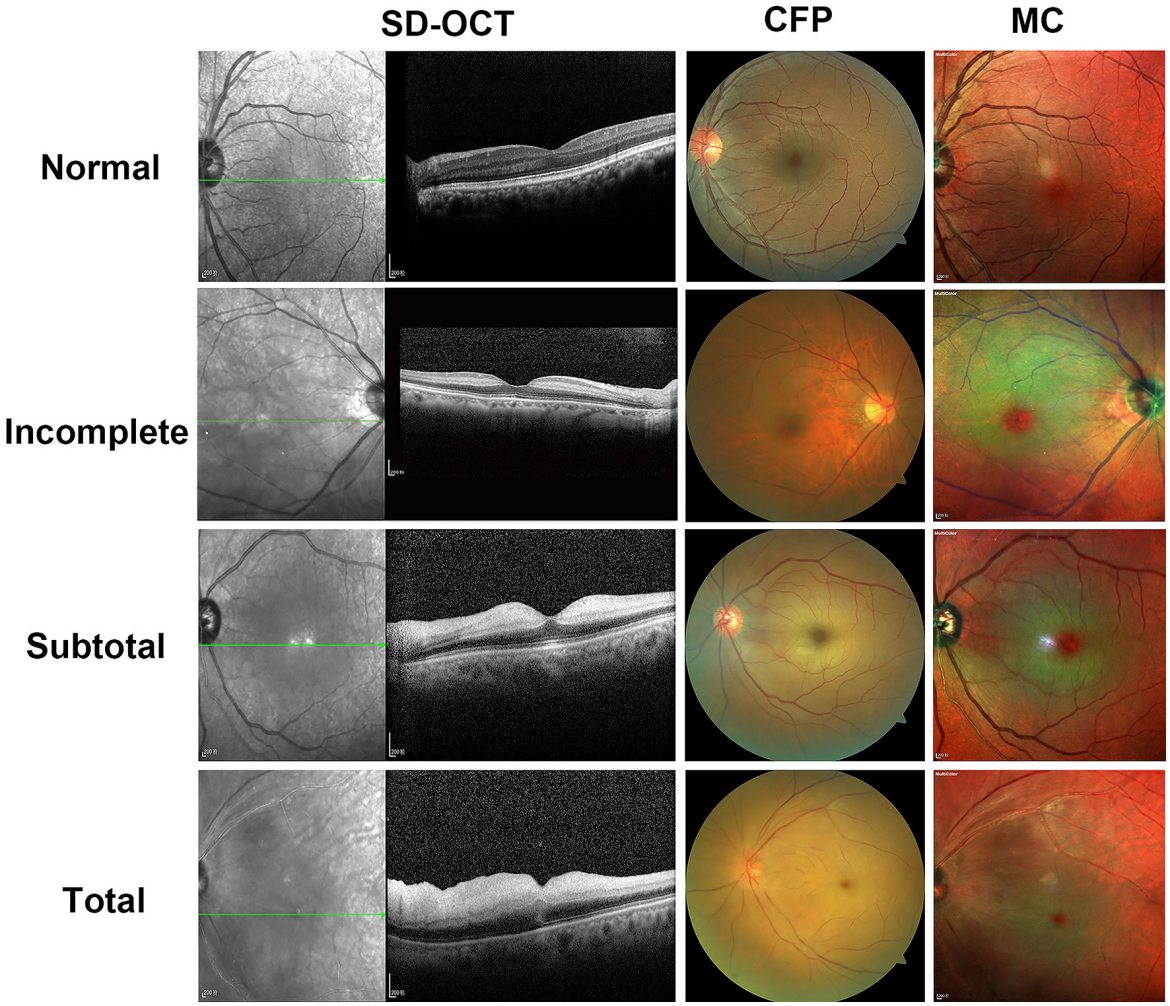
Representative photographic images of spectral-domain optical coherence tomography (SD-OCT), fundus photography (CFP), and multicolor (MC) images for incomplete, subtotal, and total acute central retinal artery occlusion (CRAO). In a 71-year-old female patient of incomplete CRAO, the VA is 2.3 logMAR; SD-OCT shows inner retinal hyperreflectivity without thickening as well as intact layer-by-layer structure. There is only slight ischemic retinal whitening on CFP. On the MC image, the edema area is a bright yellowish green reflectiveness with a transmitted orange-red color and a definite cherry-red spot. A 60-year-old man with subtotal CRAO has a VA of 2.3 logMAR and shows higher reflectivity with moderate thickness of the inner retina on SD-OCT and moderate retinal whitening on CFP. As compared with the incomplete CRAO, the color of the edema is dark green and the area of the cherry-red spot is smaller on the MC image. In a 70-year-old female patient case of total CRAO, the VA is 2.9 logMAR; hyperreflectivity of inner retinal layers, loss of organized layer structure, extreme retinal thickening, and subretinal fluid are shown on SD-OCT. The CFP appears extreme retinal edema. Grayish-green reflective area with unclear boundaries is seen on the MC image. The cherry-red spot was the smallest on the MC image as compared with subtotal CRAO or incomplete CRAO. The OCT and MC image is scaled to 1:200 μm.

Figure 1 depicts representative photographs of the incomplete, subtotal, and total CRAO for the SD-OCT, CFP, and MC images. The distinguishing SD-OCT change in the incomplete CRAO eye was inner retinal hyperreflectivity without noticeable thickening. Retinal layering is clearly visible. In the MC image, the edema area had a bright yellowish green reflectiveness with a transmitted orange-red color. A definite cherry-red spot was also detected. Only slight ischemic retinal whitening was seen on CFP. In the case of subtotal CRAO, the inner retina had higher reflectivity with moderate thickness on SD-OCT. The IPL began to appear indistinct from INL. The color was dark green, and the area of the cherry-red patch was smaller than the incomplete CRAO on the MC image. Moderate ischemic retinal edema was observed on CFP. In patients of total CRAO, significant retinal thickening and loss of organized retinal whole-layer structure were observed on SD-OCT. The IMRL to MRL of the retina exhibited hyperreflective signals. The grayish-green reflective area with unclear boundaries was found, and the cherry-red spot was the smallest on the MC image. Extreme retinal edema was found on CFP, and extensive whitening of the retina can be seen. In addition, we found MC images showed clearer retinal edema than CFPs in seven CRAO patients.

**Figure 2:**
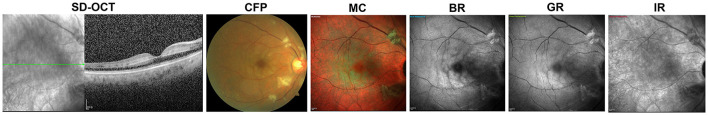
A representative case of fundus photography (CFP) misdiagnosis of central retinal artery occlusion (CRAO). The patient is a 70-year-old female with a VA of 1.4 logMAR. It had consistent spectral-domain optical coherence tomography (SD-OCT) and multicolor (MC) image grading other than CFP. The images of both SD-OCT and MC are consistent with incomplete CRAO, which is inner retinal hyperreflectivity without thickening on SD-OCT, and a bright yellowish green reflectiveness with transmitted orange-red color on MC image. However, CFP shows moderate retinal whitening, which is in accordance with the subtotal CRAO. The white hyperreflective area is on blue reflectance (BR) and green reflectance (GR) images and the dark reflection area on infrared reflectance (IR) images. The image is scaled to 1:200 μm.

The ability to grade CRAO by CFP and MC based on SD-OCT was also assessed. Among the 86 acute CRAO patients, MC image had significantly higher power of CRAO detection (80.2%) than CFP (64.0%; *P* = 0.03). Representative acute CRAO case that had the consistent SD-OCT and MC image grading other than CFP is shown in Figure 2. In this case, it was graded as incomplete CRAO on SD-OCT and MC image, while subtotal CRAO on CFP. The edema areas on BR and GR images were white hyperreflective areas with a dark hyporeflective foveal center, while on IR image were dark reflection areas with a white hyperreflective foveal center. Further analysis of the ability of grading different stages of CRAO was conducted. MC images allowed for the detection of 5 (62.5%) incomplete CRAO patients, 23 (71.9%) subtotal CRAO patients, and 41 (89.1%) total CRAO patients, respectively. Other CRAO patients were misdiagnosed with other stages of CRAO. Moreover, CFP accurately detected 2 (25.0%) incomplete CRAO cases, 19 (59.4%) subtotal CRAO cases, and 34 (73.9%) total CRAO cases, respectively. Significant differences were not found among the three stages of CRAO between MC images and CFP images (All P>0.05; Table 2). With CFP as the comparator, the results of different stages of CRAO on MC image are shown in Table 3. Among the three CRAO stages, sensitivity was highest for incomplete CRAO (100%), followed by total CRAO (94.1%), and then subtotal CRAO (68.4%) on the MC image. The specificity was low. When MC was the comparator, the sensitivity of the three stages of CRAO were low while the specificity was 100% for incomplete CRAO on CFPs (Table 4).

**Table 2:** Comparison of positive percent agreements of multicolor (MC) image and fundus photography (CFP) in different stages of acute central retinal artery occlusion (CRAO) based on spectral-domain optical coherence tomography (SD-OCT).

**Stages of CRAO**	**MC in agreement with SD-OCT (*n*, %)**	**CFP in agreement with SD-OCT (*n*, %)**	***P*-value**
Incomplete	5 (62.5)	2 (25)	0.31^a^
Subtotal	23 (71.9)	19 (59.4)	0.29
Total	41 (89.1)	34 (73.9)	0.06
All	69 (80.2)	55 (64.0)	0.03^*^

CRAO, central retinal artery occlusion; MC, multicolor; CFP, fundus photography; SD-OCT, spectral-domain optical coherence tomography.

^a^Fisher's exact test.

^*^Statistically significant across the three grades of CRAO.

**Table 3:** Results of each stage of acute central retinal artery occlusion (CRAO) comparing fundus photography (CFP) with multicolor (MC) image.

**Stages of CRAO**	**CFP**	**MC image**			
		**Consistency**	**Inconsistency**	**Sensitivity (%)**	**Specificity (%)**
Incomplete	Consistency	2	0	100.0	50.0
	Inconsistency	3	3		
Subtotal	Consistency	13	6	68.4	23.1
	Inconsistency	10	3		
Total	Consistency	32	2	94.1	25.0
	Inconsistency	9	3		

CRAO, central retinal artery occlusion; MC multicolor; CFP, fundus photography.

**Table 4:** Results of each stage of acute central retinal artery occlusion (CRAO) comparing multicolor (MC) image with fundus photography (CFP).

**Stage of CRAO**	**MC**	**CFP image**			
		**Consistency**	**Inconsistency**	**Sensitivity (%)**	**Specificity (%)**
Incomplete	Consistency	2	3	40.0	100.0
	Inconsistency	0	3		
Subtotal	Consistency	13	10	56.5	33.3
	Inconsistency	6	3		
Total	Consistency	32	9	78.0	60.0
	Inconsistency	2	3		

CRAO, central retinal artery occlusion; MC, multicolor; CFP, fundus photography.

As retinal thickness correlates with CRAO severity (Furashova and Matthé, 2017), we further calculated retinal thickness among different CRAO stages using different grading systems. In 86 acute CRAO patients, we first assessed intragrader and intergrader reliability between X.L. and Y.L. using the ICC coefficient. A value of 1.00 indicates perfect correlation, whereas a value of 0 indicates no correlation. Considering the evaluation of retinal thickness, the intergrade agreement was high for each retinal-specific layer (ICCCFP = 0.943, 95% CICFP, 0.910 to 0.963; ICC IMRL = 0.888, 95% CI IMRL 0.832 to 0.925; ICC MRL = 0.878, 95% CI MRL 0.819 to 0.919; ICC ORL = 0.943, 95% CI ORL 0.914 to 0.962). Second, we analyzed specific retinal thickness in different CRAO stages, which were graded by MC, OCT, and CFP. CFT, IMRL, MRL, and ORL thickness all had significant differences across the three acute CRAO grades (all *P* < 0.05, Table 5). *Post-hoc* Sidak's test revealed that CFT, IMRL, MRL, and ORL thickness only differed significantly in a total group compared to two other groups (*P*_CFT_ < 0.001; *P*_IMRL_ < 0.001; *P*_MRL_ < 0.001; *P*_ORL_ < 0.001). We also compared the retinal thickness of incomplete, subtotal, and total CRAO groups graded by OCT, CFP, and MC images. No statistical differences were found in CFT, IMRL, MRL, and OPL between the OCT and the MC grading groups (all *P* > 0.05, Supplementary Table 3). Between OCT and CFP grading groups, significant differences only existed in CFT (*P* = 0.019) in incomplete CRAO patients (Supplementary Table 4).

**Table 5:** Retinal thickness measurements by spectral-domain optical coherence tomography (SD-OCT) at different grades of acute central retinal artery occlusion (CRAO) identified by SD-OCT, fundus photography (CFP), and multicolor (MC).

**Retinal layer in different images/disease grades**		**Incomplete**	**Subtotal**	**Total**	***P*-value**
MC	CFT, μm	227.67 ± 29.96	233.25 ± 45.88	306.66 ± 97.34	< 0.001^*^
	IMRL, μm	105.50 ± 25.30	112.77 ± 29.62	149.44 ± 32.23	< 0.001^*^
	MRL, μm	53.61 ± 10.27	72.42 ± 22.09	93.32 ± 22.09	< 0.001^*^
	ORL, μm	168.56 ± 16.10	173.60 ± 23.19	198.20 ± 52.99	0.021^*^
OCT	CFT, μm	233.81 ± 30.43	237.30 ± 50.12	304.25 ± 98.99	0.001^*^
	IMRL, μm	119.00 ± 17.45	111.58 ± 27.20	148.55 ± 35.80	< 0.001^*^
	MRL, μm	60.94 ± 13.13	72.78 ± 23.03	91.84 ± 23.62	< 0.001^*^
	ORL, μm	167.63 ± 16.89	172.78 ± 22.17	199.36 ± 53.22	0.011^*^
CFP	CFT, μm	244.73 ± 56.87	235.65 ± 42.18	308.88 ± 101.62	< 0.001^*^
	IMRL, μm	98.92 ± 33.55	116.34 ± 24.11	153.89 ± 30.09	< 0.001^*^
	MRL, μm	59.42 ± 23.86	77.73 ± 19.14	91.88 ± 24.37	< 0.001^*^
	ORL, μm	174.77 ± 19.42	173.10 ± 22.94	200.06 ± 55.39	0.017^*^

CRAO, central retinal artery occlusion; MC, multicolor; CFP, fundus photography; SD-OCT, spectral-domain optical coherence tomography; CFT, central foveal thickness; IMRL, innermost retinal layer; MRL, middle retinal layer; ORL, outer retinal layer.

Differences between the three CRAO grades were calculated using one-way multivariate factorial ANOVA test.

^*^Statistically significant across the three grades of CRAO.

Finally, we analyzed the SCP and DCP data of CRAO patients graded by MC, OCT, and CFP. In total, 32 CRAO patients were excluded because of poor fit, low scan quality, blurred images, and shifted image uptake positions. In total, 54 patients were finally included in the analysis. There were three patients in incomplete CRAO, 24 in subtotal CRAO, and 27 in total CRAO using the OCT grading method. The proportion of the area occupied by blood flow signal in SCP of above CRAO stage were 42.48 ± 1.83, 38.79 ± 6.51, and 42.37 ± 7.84. Moreover, the mean macular DCP was 44.48 ± 7.01, 46.58 ± 6.98, and 43.11 ± 7.69, respectively. Under the CFP grading method, there were 10 incomplete CRAOs, 19 subtotal CRAOs, and 25 total CRAOs. The mean SCP in the macula were 39.12 ± 4.66, 39.70 ± 6.94, and 42.38 ± 8.00, and the mean DCP were 45.82 ± 4.80, 45.47 ± 8.38, and 43.71 ± 7.41. Under the MC grading method, there were 6 patients in incomplete CRAO, 24 in subtotal CRAO, and 24 in total CRAO. The mean macular SCP for the incomplete, subtotal, and total CRAO grades were 37.82 ± 4.71, 38.69 ± 7.15, and 43.63 ± 6.78, respectively. The proportions in DCP were 46.38 ± 4.85, 45.04 ± 8.10, and 43.94 ± 7.25 for the incomplete, subtotal, and total CRAO graded by MC, respectively. There were no significant differences in SCP and DCP in the same CRAO subgroup among three different grouping methods with OCT, CFP, or MC (Supplementary Tables 5, 6). Under the OCT and CFP grading methods, no statistical differences were found in both SCP and DCP among the three CRAO subgroups (Table 6). Significant differences existed in SCP among the three different CRAO grading groups under the MC grading system (*P* = 0.03). To our surprise, the more severe the CRAO, the higher the SCP. However, unlike SCP, the DCP was not significantly different among these three CRAOs (*P* = 0.745).

**Table 6:** SCP and DCP at different grades of CRAO identified by OCT, MC, and CFP.

**Retinal vessel density in different images/ disease grades**		**Incomplete**	**Subtotal**	**Total**	***P*-value**
OCT	SCP (%)	42.48 ± 1.83	38.79 ± 6.51	42.37 ± 7.84	0.212
	DCP (%)	44.48 ± 7.01	46.58 ± 6.98	43.11 ± 7.69	0.275
MC	SCP (%)	37.82 ± 4.71	38.69 ± 7.15	43.63 ± 6.78	0.03^*^
	DCP (%)	46.38 ± 4.85	45.04 ± 8.10	43.94 ± 7.25	0.745
CFP	SCP (%)	39.12 ± 4.66	39.70 ± 6.94	42.38 ± 8.00	0.337
	DCP (%)	45.82 ± 4.80	45.47 ± 8.38	43.71 ± 7.41	0.652

CRAO, central retinal artery occlusion; MC, multicolor; CFP, fundus photography; SD-OCT, spectral-domain optical coherence tomography; SCP, superficial capillary plexus; DCP, deep capillary plexus.

Differences between the three CRAO grades were calculated using independent-samples t-test. ^*^Statistically significant across the three grades of CRAO.

## 4 Discussion

The MC is a new imaging modality developed for SD-OCT, which can be performed with retina angiography and/or OCT simultaneously with the Spectralis instrument. It is a non-invasive technology that utilizes three laser colors penetrating and depicting information from various retinal depths. The BR image provides details of superficial structures such as RNFL and vitreoretinal interface. The GR image visualizes retinal blood vessels, exudates, and hemorrhages due to the absorption features of green wavelengths. The IR image displays layers of the outer retina, retinal pigment epithelium (RPE), and choroid as the minimal absorption of the longer wavelengths (infrared wavelength) by components. MC image compiles the three reflectances into a pseudocolor image (Tan et al., 2016). Nowadays, several studies have identified the obvious value of MC images in posterior segment pathology diseases, such as central serous chorioretinopathy (CSC; He et al., 2020; Saurabh et al., 2020), epiretinal membranes (ERM; Song et al., 2019; Terasaki et al., 2020), diabetic retinopathy (DR; Roy et al., 2019; Arrigo et al., 2021), and age-related macular degeneration (AMD; Graham et al., 2018; De Rosa et al., 2020). On MC and GR images rather than CFPs, hard exudates, cotton-wool spots, retinal microaneurysms, and hemorrhages were better identified (Roy et al., 2019; Arrigo et al., 2021).

CRAO is an emergency eye disease leading to clinically detectable retinal changes. All retinal structures, such as occluded blood vessels and ischemic edema in the inner retina, can be affected. In the late phase, CRAO will result in outer retinal thinning, photoreceptor defects, and atrophic RNFL (Ahn et al., 2015). However, so far, the application of MC image in CRAO has been less studied. No study has explored the ability of MC images to grade CRAO, and there are no reported studies that have evaluated the efficacy of MC imaging on CRAO compared with CFP, both qualitatively and quantitatively.

Retinal edema, vision loss severity, and delay in retinal perfusion were used in the previous CRAO classification system (Ahn et al., 2015). However, CFP and FA were used to grade CRAO might overlook some CRAO patients with unclear fundus signs (Gong et al., 2016). Studies showed that OCT could provide detailed morphological changes in different retinal layers at various stages of CRAO (Ahn et al., 2015; Kim et al., 2019; Matthé et al., 2020). Acute CRAO in our study were categorized as incomplete, subtotal, and total CRAO using the grading criteria suggested by Ahn et al. (2015). We therefore aimed to firstly elucidate the characteristics of different stages of CRAO using MC images and subsequently compare the CRAO grading ability between MC imaging and the most widely used fundus photographic modality, CFP, utilizing OCT-based measurement as the classification criteria. Additionally, we compared VA, retinal specific thickness, SCP, and DCP under different CRAO grading methods obtained by MC, OCT, and CFP. The feasibility of using MC for CRAO severity grading was also analyzed.

**Figure 3:**
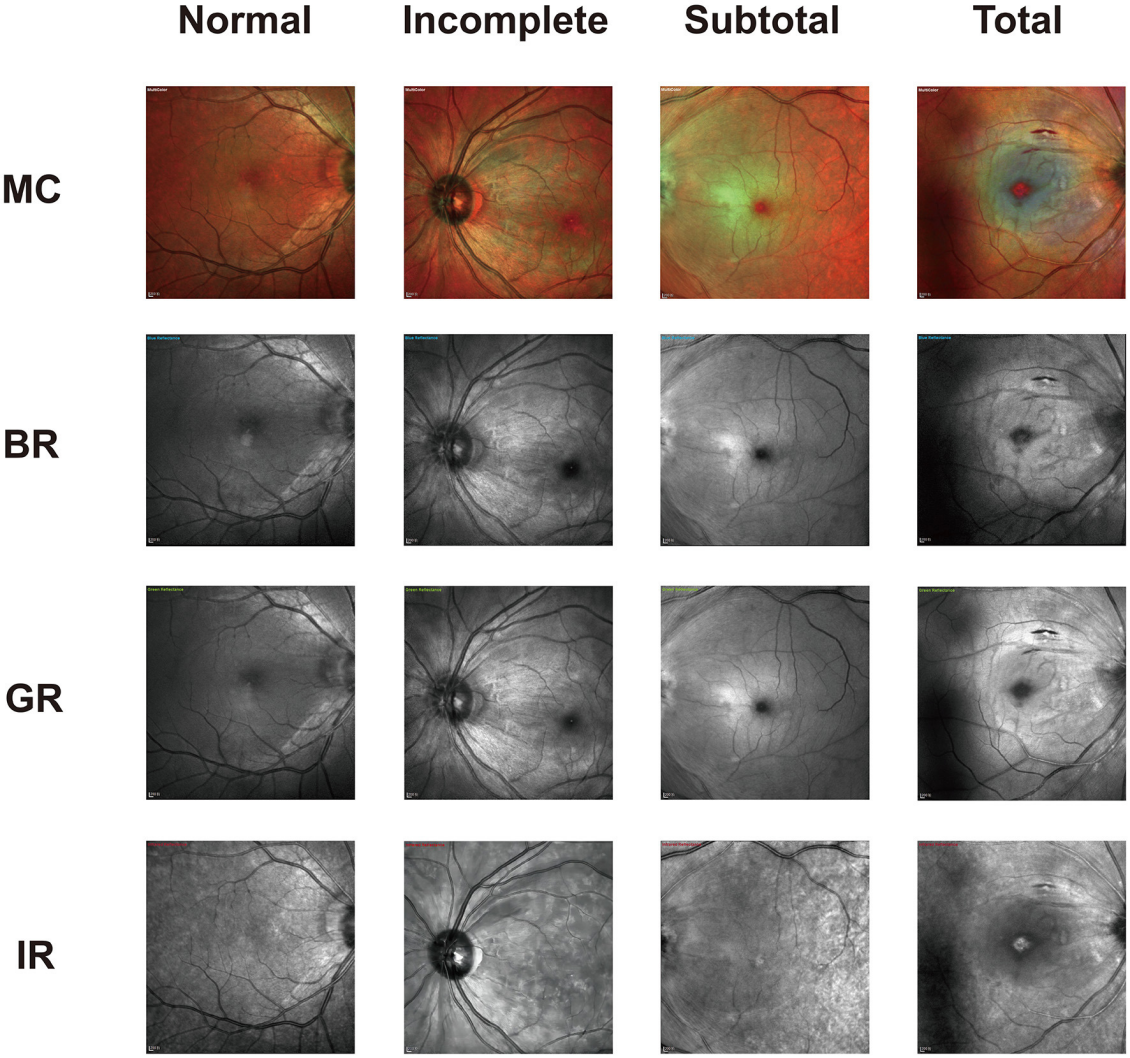
Representative photographic images of multicolor (MC) and the three-color channels for incomplete, subtotal, and total acute central retinal artery occlusion (CRAO). As displayed, both blue reflectance (BR) and green reflectance (GR) images show white hyperreflective areas in all CRAO groups. The dark reflection area on the infrared reflectance (IR) image is mostly visible in total CRAO. The image is scaled to 1:200 μm.

The distinct stages of CRAO confirmed by SD-OCT were prominently depicted on the MC image in our study. One study described acute CRAO characteristics as white areas of hyper-reflectance at the posterior pole with a normal foveal region on BR and GR and dark areas of hypo-reflectance on IR (Venkatesh et al., 2020), which is similar to our findings. However, no significant changes were observed in the pathological features of BR, GR, and IR images in patients with varying degrees of CRAO. Only in MC composite images could patients with different severity levels exhibit distinct pathological features (Figure 3). The MC image showed significantly higher sensitivity of CRAO detection than CFP. Although the positive percent agreements of grading different severity CRAO with MC image were higher than with CFPs, the differences were not significant. The small sample of each group may be the reason. More acute CRAO patients are needed to be included to analyze these results. With CFP as the reference, the sensitivity of MC images was high for all three stages of CRAOs, especially incomplete CRAO. Our study also revealed that MC images showed clearer retinal edema than CFPs and were not affected by hazy media. Moreover, MC images can be performed through a small or undilated pupil, acquiring SD-OCT images on a single machine without moving the patients to another machine.

However, studies have concluded that OCT is superior to CFP and FFA in CRAO diagnosis. Most previous studies were limited in that they included patients already diagnosed with CRAO. Subsequently, the diagnosis of CRAO was individually made based on different examination images to assess the sensitivity of these examinations (Abdellah, 2019). Many retinal diseases such as DR, AMD, and central plasma retinopathy also present with OCT imaging similar to CRAO, including macular edema, loss of retinal organized layer structure, and high reflectivity at specific retinal layers (Murthy et al., 2016). In addition, OCT only allows for longitudinal assessment of retinal layer structure, not the types of retinal artery occlusion or the extent of retinal vascular obstruction and ischemia. Therefore, it is challenging to clinically confirm the diagnosis of CRAO by a single OCT imaging presentation. Accurate diagnosis of CRAO thus requires both longitudinal and cross-sectional assessment of retinal pathologic features. MC, compared to CFP with FFA, would be a superior choice as a combination with OCT for CRAO diagnosis.

Previous studies have confirmed the clinical significance of CRAO stages, as they are associated with baseline and final VA, degrees of retinal occlusion, changes in retinal and choroidal morphology, and the selection and effectiveness of intra-arterial thrombolysis in CRAO patients (Schmidt et al., 2002; Dotan et al., 2014; Ahn et al., 2015). In our study, we analyzed VA at different CRAO grades and found that under MC grading, the more severe the CRAO obstruction, the worse the VA, consistent with previous findings. Surprisingly, there was no significant difference in VA among CRAO subgroups under OCT and CFP grading methods. However, no significant difference in VA was observed among all groups of CRAO patients between OCT and MC grading methods or when comparing OCT grading with the CFP grading method. This finding suggested that MC imaging may offer higher sensitivity for grading CRAO compared to OCT and CFP. In addition, as CRAO is an ophthalmic emergency, OCT images are needed to assess retinal reflectance and retinal thickness for grading CRAO patients. MC images can determine CRAO severity based on the color characteristics of the retinal edema area and the size of cherry erythema in a simple and intuitive manner. This can improve the efficiency of treating as well as grading CRAO patients in the clinic. Nevertheless, this conclusion requires confirmation through larger sample sizes and quantitative and qualitative studies.

The specific retinal thickness of CRAO was also analyzed in our study. Similar to the VA of CRAO patients, as the severity of CRAO increased, the retinal ischemic situation worsened. In particular, in subtotal and total CRAO, significant retinal edema was observed, and the layer-by-layer retinal structure was strongly damaged (Furashova and Matthé, 2017). No matter the grading methods, the thickness of CFT, IMRL, MRL, and ORL were significant differences among the three different CRAO groups. Despite being a qualitative measurement tool, MC can detect retinal edema by analyzing differences in the color and reflectivity of captured images. In our study, the MC grading method and OCT grading method had similar results in incomplete, subtotal, and total CRAO. This result was also comparable with the conclusions reached in previous studies: the more severe the disease, the thicker the specific retinal layer is (Furashova and Matthé, 2017). While significant differences were observed between OCT and CFP grading methods. Therefore, MC is a more accurate grading method than CFP in retinal thickness, especially in incomplete CRAO.

OCTA has facilitated a clearer delineation of the association between macular vascular density and visual outcomes. Previous studies have reported the usefulness of OCTA in non-invasively monitoring retinal circulatory dynamics following CRAO and aiding in understanding changes to the retinal circumpapillary layer (Konno et al., 2020). As the difficulty in acquiring accurate images of CRAO patients with poor cooperation and vision acuity, few studies have investigated trends in SCP and DCP at different levels of CRAO severity. Two studies have found that SCP and DCP were significantly reduced with acute CRAO (Sellam et al., 2017; Yang et al., 2019). In our study, we analyzed SCP and DCP among three CRAO subgroups graded by MC, OCT, and CFP. Compared with the OCT grading method, SCP and DCP values were not significant among the three CRAO groups in the CFP grading method nor in the MC grading method. This suggests that there is no significant difference between CFP and MC grading methods in SCP and DCP. However, our further analysis revealed that MC images appear to better reflect the trend of SCP and DCP changes with different CRAO grades. Only among MC grading images, the SCP values significantly increased with the severity of CRAO. While DCP value decreased as CRAO worsened, they did not differ significantly among the CRAO subgroups. It may be explained by the presence of both superficial and deep capillary ischemia as separate or continuous lesions occurring in the acute phase of CRAO (Yu et al., 2015). Deep capillary plexus appeared to be more involved than the superficial plexus during the acute phase of CRAO (Sellam et al., 2017). Therefore, the trends in SCP and DCP in the same patient may not correspond to each other. Moreover, preferential involvement of the deep retina results in a decreased trend of DCP, while SCP may function as a compensatory mechanism to maintain retinal blood circulation. Another reason may related to light penetration. As the severity of CRAO increases, so does the patient's retinal edema. The OCTA system used in our study has a shorter wavelength than other OCTA devices, limiting deeper tissue penetration of the light (Batioglu et al., 2023). The SCP locates from the ILM to the upper 10 μm of the IPL layer including the most severe retinal edema layer. Therefore, light penetration may also be the reason for the increased SCP values. These assumptions need to be confirmed with a larger sample.

Several limitations were presented in our study. We included a small sample size of incomplete CRAO patients, subsequent validation of the analysis with an increased sample size is needed. Second, the color of the MC image may be affected by different exposures, which made clinicians make efforts to distinguish lesions from artifacts. Meanwhile, although our analyses were performed under an objective scale by experienced ophthalmologists, evaluations of the MC images and CFP were subjective to some extent. Finally, further evaluation of parameters such as color reflectance in MC images is needed to verify the accuracy of MC images in CRAO classification.

## 5 Conclusion

In conclusion, this was the first study to qualitatively and quantitatively characterize MC images in CRAO and assess the differences in CRAO grading between CFP and MC images. Our study suggested that MC image could serve as a viable substitute for CFP in CRAO grading and screening, offering notable advantages such as being convenient, non-invasive, inexpensive, less time-consuming, more accurate, and without side effects. Translating MC examination into clinic should be a key research topic as expanding our armamentarium of tools to evaluate CRAO severity. It may help illustrate controversies around optimal therapeutics.

## Data availability statement

The original contributions presented in the study are included in the article/Supplementary material, further inquiries can be directed to the corresponding authors.

## Ethics statement

The studies involving humans were approved by Ethics Committee of Renmin Hospital of Wuhan University. The studies were conducted in accordance with the local legislation and institutional requirements. The participants provided their written informed consent to participate in this study. Written informed consent was obtained from the individual(s) for the publication of any potentially identifiable images or data included in this article.

## Author contributions

YWan: Writing – original draft. TC: Writing – review & editing. YL: Writing – review & editing. YY: Writing – original draft. YaW: Writing – original draft. YuW: Writing – original draft. XL: Writing – original draft. AY: Writing – review & editing. XX: Writing – review & editing.
