# Supplementary material for: Clinical application of multicolor scanning laser ophthalmology in diagnosis and grading of central retinal artery occlusion
Source: Front Neurosci. 2024 Apr 10;18:1327806. doi: 10.3389/fnins.2024.1327806 (PMC11039857; doi:10.3389/fnins.2024.1327806)
Supplement: Supplementary file 1 [file Data_Sheet_1.docx]

Supplementary Material

# Supplementary Figure

## Supplementary Table 1. Comparsion of VA in CRAO patients with same disease severity in OCT and MC images.

| **Disease Grade/VA in different images** | **OCT** | **MC** | **P Value** |
| --- | --- | --- | --- |
| **incomplete** | 2.1±0.5 | 2.0±0.5 | 0.383 |
| **subtotal** | 2.3±0.4 | 2.4±0.3 | 0.581 |
| **total** | 2.5±0.4 | 2.4±0.4 | 0.412 |

VA= visual acuity; CRAO= central retinal artery occlusion; MC= multicolor; CFP= fundus photography; SD-OCT= spectral-domain optical coherence tomography.

## Comparsion of VA in CRAO patients with same disease severity in OCT and CFP images.

| **Disease Grade/VA in different images** | **OCT** | **CFP** | **P Value** |
| --- | --- | --- | --- |
| **incomplete** | 2.1±0.5 | 2.4±0.3 | 0.472 |
| **subtotal** | 2.3±0.4 | 2.3±0.4 | 0.7 |
| **total** | 2.5±0.4 | 2.4±0.4 | 0.49 |

VA= visual acuity; CRAO= central retinal artery occlusion; MC= multicolor; CFP= fundus photography; SD-OCT= spectral-domain optical coherence tomography.

## Comparsion of selected retinal thickness in CRAO patients with same disease severity in OCT and MC images.

| **Disease Grade/Retinal Layer in different images** | | **OCT** | **MC** | **P Value** |
| --- | --- | --- | --- | --- |
| **incomplete** | **CFT,µm** | 233.81±30.43 | 227.67±29.96 | 0.909 |
|  | **IMRL,µm** | 119.00±17.45 | 105.50±25.30 | 0.621 |
|  | **MRL.µm** | 60.94±13.13 | 53.61±10.27 | 0.386 |
|  | **ORL,µm** | 167.63±16.89 | 168.56±16.10 | 0.918 |
| **subtotal** | **CFT,µm** | 237.30±50.12 | 233.25±45.88 | 0.665 |
|  | **IMRL,µm** | 111.58±27.20 | 112.77±29.62 | 0.920 |
|  | **MRL.µm** | 72.78±23.03 | 72.42±22.09 | 0.584 |
|  | **ORL,µm** | 172.78±22.17 | 173.60±23.19 | 0.954 |
| **total** | **CFT,µm** | 304.25±98.99 | 304.25±98.99 | 0.939 |
|  | **IMRL,µm** | 148.55±35.80 | 148.55±35.80 | 0.509 |
|  | **MRL.µm** | 91.84±23.62 | 91.84±23.62 | 0.727 |
|  | **ORL,µm** | 199.36±53.22 | 199.36±53.22 | 0.945 |

CRAO= central retinal artery occlusion; MC= multicolor; SD-OCT= spectral-domain optical coherence tomography;CFT=central foveal thickness; IMRL=innermost retinal layer; MRL=middle retinal layer; ORL=outer retinal layer. Differences between the three CRAO grades were calculated using independent samples T test.

* Statistically signifificant across the three grades of CRAO.

## Supplementary Table 2. Comparsion of selected retinal thickness in CRAO patients with same disease severity in OCT and CFP images.

| **Disease Grade/Retinal Layer in different images** | | **OCT** | **CFP** | **P Value** |
| --- | --- | --- | --- | --- |
| **incomplete** | **CFT,µm** | 233.81±30.43 | 244.73±56.87 | **0.019** |
|  | **IMRL,µm** | 119.00±17.45 | 98.92±33.55 | 0.056 |
|  | **MRL.µm** | 60.94±13.13 | 59.42±23.86 | 0.110 |
|  | **ORL,µm** | 167.63±16.89 | 174.77±19.42 | 0.794 |
| **subtotal** | **CFT,µm** | 237.30±50.12 | 235.65±42.18 | 0.341 |
|  | **IMRL,µm** | 111.58±27.20 | 116.34±24.11 | 0.525 |
|  | **MRL.µm** | 72.78±23.03 | 77.73±19.14 | 0.175 |
|  | **ORL,µm** | 172.78±22.17 | 173.10±22.94 | 0.853 |
| **total** | **CFT,µm** | 304.25±98.99 | 308.88±101.62 | 0.784 |
|  | **IMRL,µm** | 148.55±35.80 | 153.89±30.09 | 0.462 |
|  | **MRL.µm** | 91.84±23.62 | 91.88±24.37 | 0.736 |
|  | **ORL,µm** | 199.36±53.22 | 200.06±55.39 | 0.803 |

CRAO= central retinal artery occlusion; CFP= fundus photography; SD-OCT= spectral-domain optical coherence tomography;CFT=central foveal thickness; IMRL=innermost retinal layer; MRL=middle retinal layer; ORL=outer retinal layer. Differences between the three CRAO grades were calculated using independent samples T test.

* Statistically signifificant across the three grades of CRAO.

## Supplementary Table 3. Comparsion of superficial capillary plexus (SCP) and deep capillary plexus (DCP) in CRAO patients with same disease severity in OCT and CFP images.

| **Retinal vessel density in CRAO/Grading method** | | **OCT** | **CFP** | **P Value** |
| --- | --- | --- | --- | --- |
|  |  |  |  |  |
| **SCP**(%) | **incomplete** | 42.48±1.83 | 39.12±4.66 | 0.108 |
|  | **subtotal** | 38.79±6.51 | 39.70±6.94 | 0.591 |
|  | **total** | 42.37±7.84 | 42.38±8.00 | 0.988 |
| **DCP**(%) | **incomplete** | 44.48±7.01 | 45.82±4.80 | 0.612 |
|  | **subtotal** | 46.58±6.98 | 45.47±8.38 | 0.516 |
|  | **total** | 43.11±7.69 | 43.71±7.41 | 0.919 |

CRAO= central retinal artery occlusion; MC= multicolor; CFP= fundus photography; SD-OCT= spectral-domain optical coherence tomography; SCP=superficial capillary plexus;DCP=deep capillary plexus. Differences between the three CRAO grades were calculated using independent samples T test.

## Supplementary Table 4. Comparsion of superficial capillary plexus (SCP) and deep capillary plexus (DCP) in CRAO patients with same disease severity in OCT and MC images.

| **Retinal vessel density in CRAO/Grading method** | | **OCT** | **MC** | **P Value** |
| --- | --- | --- | --- | --- |
|  |  |  |  |  |
| **SCP**(%) | **incomplete** | 42.48±1.83 | 37.82±4.71 | 0.165 |
|  | **subtotal** | 38.79±6.51 | 38.69±7.15 | 0.582 |
|  | **total** | 42.37±7.84 | 43.63±6.78 | 0.481 |
| **DCP**(%) | **incomplete** | 44.48±7.01 | 46.38±4.85 | 0.711 |
|  | **subtotal** | 46.58±6.98 | 45.04±8.10 | 0.619 |
|  | **total** | 43.11±7.69 | 43.94±7.25 | 0.693 |

CRAO= central retinal artery occlusion; MC= multicolor; CFP= fundus photography; SD-OCT= spectral-domain optical coherence tomography; SCP=superficial capillary plexus;DCP=deep capillary plexus. Differences between the three CRAO grades were calculated using independent samples T test
